# Assessment of left ventricular tissue mitochondrial bioenergetics in patients with stable coronary artery disease

**DOI:** 10.1038/s44161-023-00312-z

**Published:** 2023-08-07

**Authors:** Richard E. Jones, Anja V. Gruszczyk, Christina Schmidt, Daniel J. Hammersley, Lukas Mach, Michael Lee, Joyce Wong, Ming Yang, Suzan Hatipoglu, Amrit S. Lota, Sam N. Barnett, Rebecca Toscano-Rivalta, Ruth Owen, Shahzad Raja, Fabio De Robertis, Hassiba Smail, Anthony De-Souza, Ulrich Stock, Peter Kellman, Julian Griffin, Marc-Emmanuel Dumas, Jack L. Martin, Kourosh Saeb-Parsy, Ali Vazir, John G. F. Cleland, Dudley J. Pennell, Sunil K. Bhudia, Brian P. Halliday, Michela Noseda, Christian Frezza, Michael P. Murphy, Sanjay K. Prasad

**Affiliations:** 1 National Heart and Lung Institute, Imperial College London, London, UK; 2 Royal Brompton and Harefield Hospitals, Guy’s and St. Thomas’ NHS Foundation Trust, London, UK; 3 Anglia Ruskin University, Chelmsford, UK; 4 Essex Cardiothoracic Centre, Basildon, UK; 5 MRC Mitochondrial Biology Unit, University of Cambridge, Cambridge, UK; 6 MRC Cancer Unit, University of Cambridge, Cambridge, UK; 7 University of Cologne, CECAD, Cologne, Germany; 8 Department of Medical Statistics, London School of Hygiene and Tropical Medicine, London, UK; 9 National Heart, Lung, and Blood Institute, National Institutes of Health, Bethesda, MD USA; 10 The Rowett Institute, University of Aberdeen, Aberdeen, UK; 11 Department of Metabolism, Digestion and Reproduction, Imperial College London, London, UK; 12 European Genomic Institute of Diabetes, INSERM U1283, CNRS 8199, Institut Pasteur de Lille, Lille University Hospital, University of Lille, Lille, France; 13 McGill Genome Centre, McGill University, Montréal, QC Canada; 14 Department of Surgery and Cambridge NIHR Biomedical Research Centre, Biomedical Campus, University of Cambridge, Cambridge, UK; 15 Robertson Centre for Biostatistics, University of Glasgow, Glasgow, UK

**Keywords:** Translational research, Cardiovascular biology, Acute coronary syndromes, Energy metabolism

## Abstract

Recurrent myocardial ischemia can lead to left ventricular (LV) dysfunction in patients with coronary artery disease (CAD). In this observational cohort study, we assessed for chronic metabolomic and transcriptomic adaptations within LV myocardium of patients undergoing coronary artery bypass grafting. During surgery, paired transmural LV biopsies were acquired on the beating heart from regions with and without evidence of inducible ischemia on preoperative stress perfusion cardiovascular magnetic resonance. From 33 patients, 63 biopsies were acquired, compared to analysis of LV samples from 11 donor hearts. The global myocardial adenosine triphosphate (ATP):adenosine diphosphate (ADP) ratio was reduced in patients with CAD as compared to donor LV tissue, with increased expression of oxidative phosphorylation (OXPHOS) genes encoding the electron transport chain complexes across multiple cell types. Paired analyses of biopsies obtained from LV segments with or without inducible ischemia revealed no significant difference in the ATP:ADP ratio, broader metabolic profile or expression of ventricular cardiomyocyte genes implicated in OXPHOS. Differential metabolite analysis suggested dysregulation of several intermediates in patients with reduced LV ejection fraction, including succinate. Overall, our results suggest that viable myocardium in patients with stable CAD has global alterations in bioenergetic and transcriptional profile without large regional differences between areas with or without inducible ischemia.

## Abstract (web summary)

A single-center, observational cohort study assessing the mitochondrial bioenergetics of left ventricular tissue from patients undergoing coronary artery bypass grafting surgery suggests that viable myocardium has global alterations in bioenergetics and transcriptome without large regional differences between areas with or without inducible ischemia.

## Main

Despite successful implementation of population-level strategies to reduce the burden of coronary artery disease (CAD), recent increases in obesity and diabetes are driving a surge in the incidence of atherosclerosis^1^. Additionally, the advent of rapid reperfusion strategies for acute myocardial infarction, alongside the adoption of evidence-based medical therapies, has resulted in declining case fatality rates and a subsequent increase in the global burden of chronic coronary syndromes^1^. Compounding the issue of rising disease burden, there remain gaps in evidence regarding the optimal treatment strategy for patients with stable CAD^2^, notably pertaining to the role of coronary revascularization. A key objective of restoring epicardial blood flow is to increase tissue perfusion during periods of increased demand to improve symptoms and, at least conceptually, to protect the myocardium from the deleterious effects of ischemia that might lead to myocardial dysfunction and heart failure. Importantly, however, there remains a lack of human data detailing abnormalities in myocardial cellular function associated with myocardial ischemia. This includes changes to energy availability (in the form of adenosine triphosphate (ATP):adenosine diphosphate (ADP) ratio) and alterations in oxidative phosphorylation (OXPHOS). Furthermore, it remains unclear whether metabolites that drive the maladaptive response to severe hypoxia (for example, the tricarboxylic acid (TCA) cycle intermediate, succinate^2,3^) accumulate within human myocardium exposed to repetitive episodes of transient ischemia. The paucity of data arises, in part, from the lack of access to relevant human cardiac tissue. Previous studies in patients with advanced heart failure, where tissue is more readily available, have highlighted impairment of myocardial energetics^4–6^. However, whether this generalizes to the larger group of patients with relatively preserved left ventricular (LV) function is unclear.

To explore this issue further, we integrated information from quantitative perfusion cardiovascular magnetic resonance (CMR) before elective coronary artery bypass grafting (CABG) with metabolomic and transcriptomic analyses of LV biopsies from myocardial regions with and without inducible ischemia obtained at the time of surgery. This study aimed to assess for cumulative and long-term myocardial adaptations in patients with stable CAD.

## Results

**Fig. 1:**
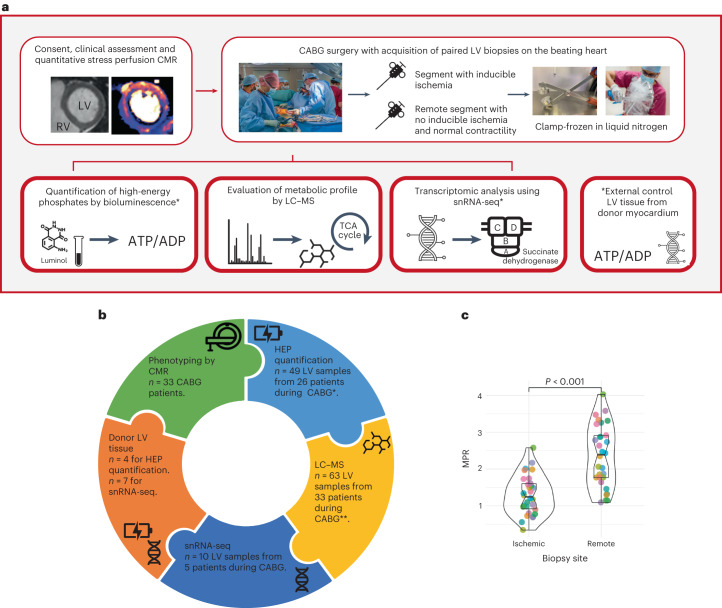
Study overview, workflow and CMR results. **a**, Diagram outlining the experimental workflow of the AMBITION study. Consented patients underwent a full clinical evaluation, including quantitative stress perfusion CMR. Guided by the CMR results, paired LV biopsies from areas with and without inducible ischemia were acquired on the beating heart during CABG. All samples were rapidly clamp-frozen in liquid nitrogen within theater. The myocardial biopsies were analyzed using up to three methods: (1) HEP quantification using a luciferin/luciferase-based bioluminescence assay; (2) metabolomic analysis by LC–MS; and (3) transcriptomic analysis by snRNA-seq. **b**, Summary of the laboratory analyses undertaken on the myocardial biopsies. The a priori aim was to acquire paired LV biopsies from regions with (‘ischemic’ biopsy) and without (‘remote’ biopsy) inducible myocardial ischemia, permitting HEP quantification and LC–MS in all patients. Surgical discretion and insufficient sample resulted in a lack of HEP and LC–MS data in some patients. Where it was deemed safe, additional myocardium was acquired from the same sites for snRNA-seq. **c**, Violin box plots demonstrating a significant difference in the biopsy site MPR from regions with and without preoperative inducible ischemia, calculated by quantitative stress perfusion CMR from 32 patients. Each color represents an individual patient. Data are presented as mean ± s.d., analyzed using a paired *t*-test^†^. LV biopsies from human heart donors were used as a control dataset in the HEP and snRNA-seq arms. * For the paired analysis of ischemic versus remote biopsies, 46 LV samples from 23 patients are presented in the HEP results. ** For the paired analysis of ischemic versus remote biopsies, 58 LV samples from 29 patients are presented in the LC–MS results.

From 33 patients (mean age 60 ± 9 years, 31 men (94%), median left ventricular ejection fraction (LVEF) 67% (interquartile range (IQR): 61–71%)), 63 LV biopsies were obtained during CABG from sites with or without inducible ischemia on preoperative stress perfusion CMR (Extended Data Table 1). Surgical discretion resulted in three patients not having paired biopsies. A summary of the experiments is outlined in Fig. 1a,b, and a full breakdown of the analyses performed on each patient is detailed in Extended Data Table 2.

The mean global myocardial perfusion reserve (MPR) was 1.8 ± 0.5, with a significant difference in segmental MPR of LV regions with and without inducible ischemia (mean ± s.d.: 1.3 ± 0.5 and 2.3 ± 0.8, respectively, *P* < 0.001; Fig. 1c). Of the 33 patients, 26 (79%) CABG operations were performed off-pump without cardiopulmonary bypass, cardioplegia or therapeutic hypothermia. The remaining seven (21%) patients underwent biopsy on the beating heart before aortic cross-clamp and cardioplegia. The metadata for the human donors are detailed in Extended Data Table 3.

### Global myocardial bioenergetic landscape in patients with stable CAD

**Fig. 2:**
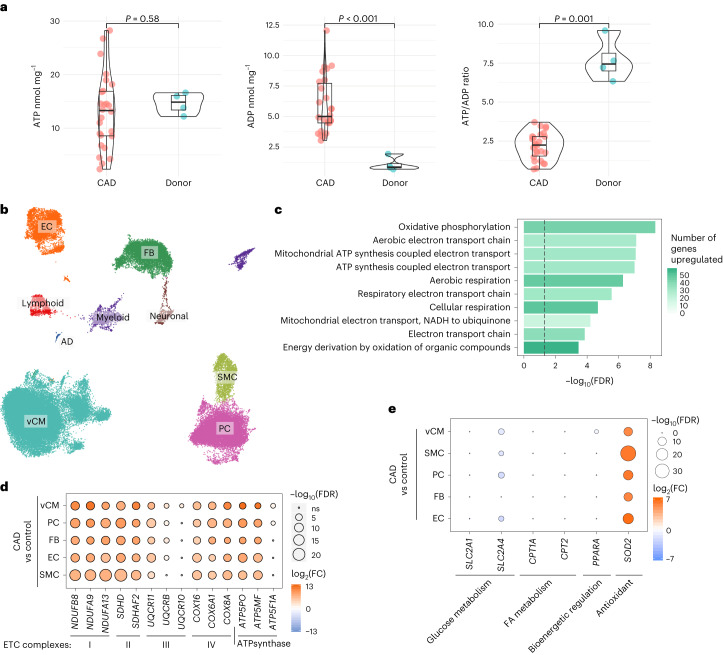
Global alterations in LV HEP content and single-nucleus transcriptomic landscape between patients with CAD and control donor myocardium. **a**, Violin box plots demonstrating the global myocardial HEP content of 26 patients with stable CAD versus donor control myocardium from four individuals, analyzed and quantified using a luciferase-based bioluminescence assay. The results demonstrate a lower ATP:ADP ratio in CAD biopsies as compared to the donor heart samples. The violin box plots visualize the distribution of the data, alongside the median value, hinges corresponding to the first and third quartiles, and whiskers corresponding to the largest/smallest value no farther than 1.5× IQR from the hinge. Analyzed using the Wilcoxon–Mann–Whitney *U*-test. **b**, UMAP representation of 65,886 nuclei from five pairs of CABG samples and seven donor controls, color-coded by cell type. **c**, Gene Ontology analysis of the snRNA-seq data demonstrating upregulation of several biological processes associated with normal cellular bioenergetic function within vCMs in patients undergoing CABG compared to control donor myocardium. The *x* axis shows the significance level, represented as −log_10_(FDR), and the color indicates the number of upregulated genes that match the Gene Ontology term. The dashed line indicates the significance threshold of FDR 0.05. **d**, Differential single-nucleus transcriptomic expression of genes associated with OXPHOS; CABG patients versus controls, stratified by cell type. The results detailing increased expression of genes encoding ETC complexes and ATP synthase in patients with CAD compared to controls, broadly aligned across cell types. **e**, Differential expression of genes (identified by snRNA-seq) implicated in glucose metabolism, fatty acid metabolism, bioenergetic regulation and endogenous antioxidant mechanisms; CABG versus control samples stratified by cell type. AD, adipocytes; NADH, nicotinamide adenine dinucleotide; FA, fatty acid; NS, non-significant.

Global high-energy phosphate (HEP) content of viable LV myocardium from 26 patients with CAD was assessed, comparing with results of four control LV samples acquired on the beating heart after brainstem death^7^. On univariate analysis, no significant difference was observed in myocardial ATP content between groups (median (IQR), nmol mg^−1^: 13.3 (8.5–16.9) and 14.9 (13.0–16.3), respectively, *P* = 0.58; Fig. 2a); however, there was a significant increase in myocardial ADP concentrations in the patients with CAD versus donor controls (median (IQR), nmol mg^−1^: 5.0 (4.5–7.9) and 0.9 (0.8–1.4), respectively, *P* < 0.001; Fig. 2a). Consequently, the ATP:ADP ratio was significantly reduced in patients with CAD as compared to donor control samples (median (IQR): 2.2 (1.5–2.8) versus 7.4 (6.8–8.6), *P* < 0.001; Fig. 2a).

Single-nucleus RNA sequencing (snRNA-seq) was performed on 10 LV samples from five patients to assess patterns of gene expression, comparing to data from seven donor LV samples acquired after brainstem (*n* = 6) or circulatory (*n* = 1) death^8^. Uniform manifold approximation and projection (UMAP) representation of the isolated nuclei is presented in Fig. 2b. Gene Ontology analysis highlighted upregulation of several biological processes associated with normal cellular respiration and OXPHOS in patients with CAD compared to donor myocardium (Fig. 2c). Specifically, genes associated with the mitochondrial electron transport chain (ETC) complex I (including *NDUFA13*, *NDUFA9* and *NDUFB8*), complex II (*SDHAF2* and *SDHD*), complex III (including *UQCR10*, *UQCR11* and *UQCRB*), complex IV (including *COX16*, *COX6A1* and *COX8A*) and ATP synthase (including *ATP5F1A*, *ATP5MF* and *ATP5PO*) had increased expression within LV cardiomyocytes of patients with CAD compared to donor control samples (Fig. 2d). Furthermore, expression of these key genes broadly aligned across the other cell types, including fibroblasts (FBs), endothelial cells (ECs), pericytes (PCs) and smooth muscle cells (SMCs) (Fig. 2d). There was increased expression of *SOD2*, the gene encoding manganese-dependent superoxide dismutase (MnSOD), in patients with CAD compared to controls across all cell types (Fig. 2e). Expression of *SLC2A4*, the gene encoding the insulin-sensitive glucose transporter GLUT4, was significantly reduced across multiple cell types in CABG samples compared to donor myocardium (Fig. 2e). There was also reduced expression of *PPARA*, the gene encoding PPAR-α, in ventricular cardiomyocytes (vCMs) of patients with CAD compared to controls (Fig. 2e). Expression of *CPT1A* and *CPT2*, the genes encoding carnitine palmitoyltransferase 1A and carnitine palmitoyltransferase 2, respectively, were similar between CAD and controls (Fig. 2e). Gene specificity plots, cell abundance plots and UMAP embedding of the nuclei are presented in Extended Data Fig. 2. Additionally, volcano plots of differential gene expression between groups is presented in Extended Data Figs. 3–5, and metabolomic and transcriptomic correlation data are shown in the Supplementary Data Table.

### Regional myocardial bioenergetic profile in patients with stable CAD

**Fig. 3:**
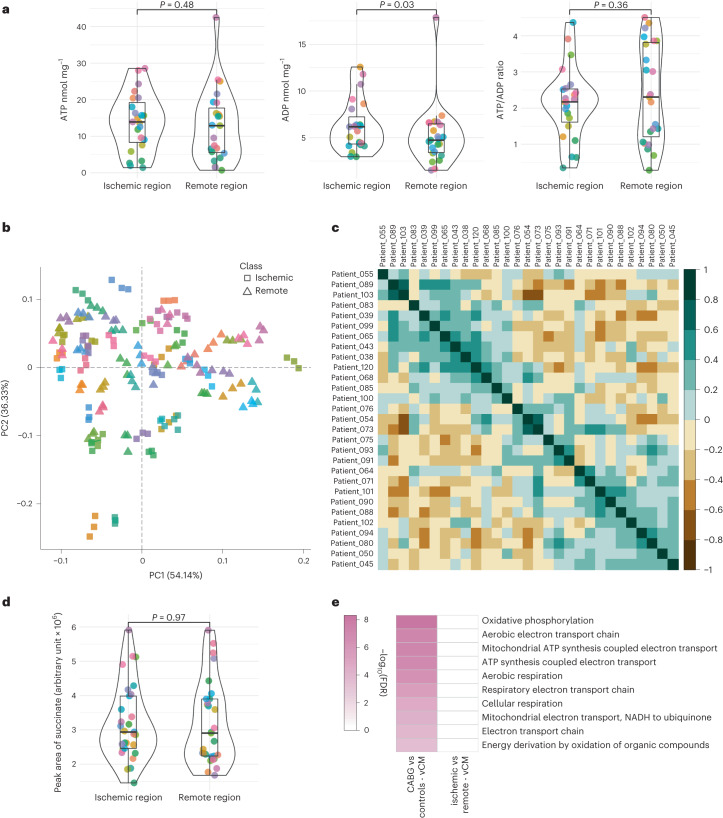
Regional differences in LV metabolic profile in patients with stable CAD. **a**, Violin box plots demonstrating paired analysis of the myocardial HEP content in 23 patients with preoperative evidence of regional myocardial ischemia, quantified using a luciferase-based bioluminescence assay. Each color represents an individual patient. The results show that myocardial ATP levels and the ATP:ADP ratio are not different between regions with and without inducible ischemia from within the same LV. Analyzed using the Wilcoxon signed-rank test. **b**, PCA of the LC–MS data revealing no clusters based on ischemic (square) or remote (triangle) biopsies and no clear clusters of patients. Each color represents an individual patient, with the three analytical repeats from each patient included in the plot. **c**, log_2_FC of the LC–MS differential metabolite analysis (comparing ischemic versus remote samples) with hierarchical clustering. The metabolite correlation between patients within those clusters is relatively minor (predominantly 20–50%), suggesting that the patterns of regional metabolite changes within individuals are not significantly replicated across the cohort. **d**, Violin box plots detailing paired univariate analysis of succinate (assessed by LC–MS), comparing regions of chronic ischemia and remote myocardium from 29 patients. The result demonstrated no significant difference in the level of this intermediate between regions. Analyzed using the Wilcoxon signed-rank test **e**, Gene Ontology analysis of the snRNA-seq data demonstrating no significant differential expression of OXPHOS genes in vCMs between ischemic and remote segments from within the same LV. CABG versus control sample results are included as a reference. The violin box plots visualize the distribution of the data, alongside the median value, hinges corresponding to the first and third quartiles and whiskers corresponding to the largest/smallest value no farther than 1.5× IQR from the hinge. FA, fatty acid; NADH, nicotinamide adenine dinucleotide; PC, principal component.

Overall, 46 paired LV samples (that is, LV biopsies from areas with and without inducible ischemia in the same heart) from 23 patients were included for the regional assessment of HEP content. Paired univariate analysis revealed no significant difference in concentrations of ATP (median (IQR), nmol mg^−1^: 13.9 (7.6–20.4) versus 12.9 (5.4–19.2), *P* = 0.48) or the ATP:ADP ratio (median (IQR): 2.2 (1.5–2.5) versus 2.3 (1.1–3.9), *P* = 0.36) between regions with and without inducible ischemia (Fig. 3a). The myocardial ADP content was slightly higher in regions with inducible ischemia compared to regions without inducible ischemia (median (IQR), nmol mg^−1^: 6.2 (4.2–8.1) versus 4.7 (3.3–6.6), respectively, *P* = 0.03; Fig. 3a).

On metabolite profiling by liquid chromatography–mass spectrometry (LC–MS), 58 paired LV samples from 29 patients were included in the final analysis. Principal component analysis (PCA) revealed no clear patient clusters nor a separation of samples originating from myocardium with and without inducible ischemia (Fig. 3b). Furthermore, differential metabolite analysis of each patient (comparing results from myocardium with and without inducible ischemia) revealed that individual alterations in metabolic landscape do not correlate well between patients, with a resultant paucity of strong patient clusters (Fig. 3c).

Specifically assessing metabolites known to accumulate in acute ischemia^2,7^, there was no significant difference in the levels of succinate (median (IQR), arbitrary units: 2.9 (2.5–4.0) versus 2.9 (2.2–3.9), *P* = 0.97; Fig. 3d), lactate (median (IQR), arbitrary units: 12.3 (8.3–19.3) versus 12.1 (7.8–18.4), *P* = 0.62; Extended Data Fig. 6a) or hypoxanthine (median (IQR), arbitrary units: 2.2 (1.3–4.2) versus 2.2 (1.2–3.3), *P* = 0.54; Extended Data Fig. 6a) between regions with or without inducible ischemia. On assessment of substrate content between regions, there was no significant difference in levels of glucose (*P* = 0.44) and the glycolytic intermediates dihydroxyacetone phosphate (*P* = 0.69) and phosphoenolpyruvate (*P* = 0.88) (Extended Data Fig. 6b).

In the snRNA-seq arm, 10 paired samples from five patients were included. Gene Ontology analysis revealed no significant differential expression of biological processes pertaining to OXPHOS between areas with and without inducible ischemia within vCMs (Fig. 3e). Additionally, there was no significant difference in the expression of genes implicated in glucose transport (*SLC2A1* and *SLC2A4*), fatty acid metabolism (*CPT1A* and *CPT2*) or regulation of cellular bioenergetic function (*PPARA*, *PRKAA2* and *PPARGC1A*) between regions with or without inducible ischemia in vCMs (Extended Data Fig. 2e).

### Exploratory analysis assessing LV metabolism in stable CAD

**Fig. 4:**
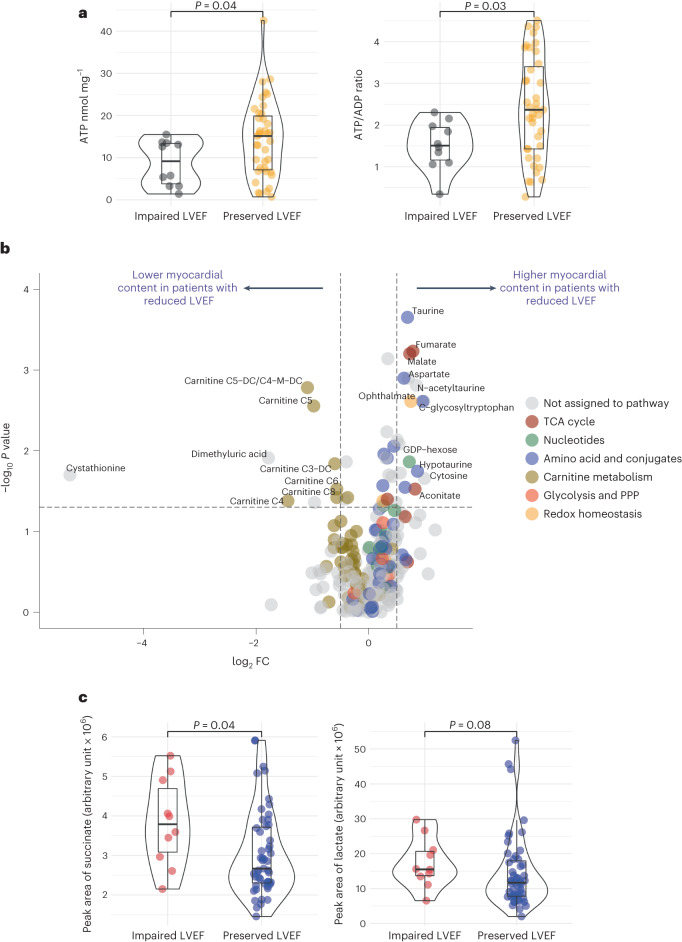
Exploratory analysis comparing the bioenergetic profile of patients with CAD with preserved LVEF versus impaired LVEF. **a**, Exploratory univariate analysis of myocardial HEP content in 26 patients with CAD, stratifying by LV function, analyzed and quantified using a luciferase-based bioluminescence assay. The results demonstrate a significant reduction in ATP levels and the ATP:ADP ratio in patients with impaired LVEF compared to preserved LVEF. **b**, Volcano plot of the LC–MS data highlighting metabolites that were significantly altered between patients with and without reduced LVEF (*P* < 0.05 with log_2_FC > 0.5 or log_2_FC < −0.5). **c**, Exploratory univariate analysis of the LC–MS data in 29 patients, the results suggesting elevated succinate levels in patients with CAD with reduced LVEF compared to patients with preserved LVEF. The violin box plots visualize the distribution of the data, alongside the median value, hinges corresponding to the first and third quartiles and whiskers corresponding to the largest/smallest value no farther than 1.5× IQR from the hinge. In these exploratory experiments, biopsies from regions with and without preoperative inducible ischemia were individually included as biological replicates. DC, dicarboxy; DV, dysfunctional/hypocontractile but viable myocardium; PPP, pentose phosphate pathway.

Subgroup analysis was performed on the HEP and LC–MS results, initially stratifying patients by LVEF. In this exploratory analysis, patients with paired biopsies had both samples included as biological replicates. Of the 26 patients in the HEP analysis, six (23%) patients had reduced LVEF according to the normal ranges adjusted to age, sex and body surface area (median LVEF (IQR): 47% (43–54%) versus 68% (66–71%))^9^. There was a significant reduction in global myocardial ATP levels (median (IQR), nmol mg^−1^: 9.2 (3.3–13.5) versus 15.1 (6.8–20.4), *P* = 0.04) and the ATP:ADP ratio (median (IQR), 1.5 (1.1–2.0) versus 2.4 (1.4–3.5), *P* = 0.03) in patients with reduced LVEF as compared to patients with preserved LVEF (Fig. 4a). There was no significant difference in myocardial ADP content in patients with impaired LV function compared to patients with preserved function (median (IQR), nmol mg^−1^: 5.3 (3.6–6.7) versus 5.1 (4.2–6.6) respectively, *P* = 0.78).

Metabolomic analysis included six (23%) patients with reduced LVEF (median LVEF (IQR): 43% (41–49%) versus 68% (66–72%)). Differential metabolite analysis revealed 17 metabolites that were significantly upregulated (log_2_(fold change (FC)) > 0.5 and *P* < 0.05) in patients with impaired compared to preserved LVEF, including multiple amino acids (for example, taurine, hypotaurine and isoleucine) in addition to several TCA cycle metabolites (Fig. 4b and Supplementary Data Table). Additionally, eight metabolites were significantly downregulated (log_2_FC < −0.5 and *P* < 0.05) in patients with impaired LVEF compared to preserved LVEF, notably several short-chain and medium-chain acylcarnitines (Fig. 4b and Supplementary Data Table).

Given our a priori hypotheses pertaining to metabolites that accumulate in acute ischemia^2^, we performed further analysis on these intermediates. In this analysis, there was a significant increase in myocardial succinate (median (IQR), arbitrary units: 3.8 (3.0–4.9) versus 2.7 (2.3–3.7), *P* = 0.04) and a similar trend for lactate (median (IQR), arbitrary units: 15.5 (13.5–21.0) versus 11.7 (7.8–18.4), *P* = 0.08) in patients with CAD with a reduced compared to preserved LVEF (Fig. 4c,d). Extended data are presented in the Supplementary Data Table and Extended Data Figs. 6c and 7.

## Discussion

**Extended Data Fig. 1:**
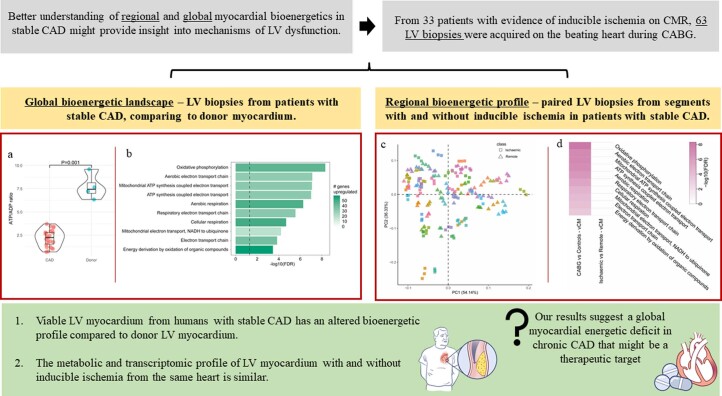
Summary of the study findings. Patients undergoing CABG had LV biopsies acquired on the beating heart. LV samples from donor hearts were included as controls. First, global alterations between CAD patients and control donor myocardium were assessed. **a:** Violin box-plot demonstrating the lower ATP/ADP ratio in CAD biopsies as compared to donor samples. **b:** Gene ontology analysis demonstrating upregulation of several biological processes associated with normal cellular function within ventricular cardiomyocytes of CAD patients compared to donor myocardium. Second, regional differences in LV metabolic profile were assessed, using paired biopsies from segments with and without inducible ischaemia (as determined by CMR). **c:** Principal component analysis of the liquid chromatography–*mass spectrometry* data revealing no clusters based on ischaemic (square) or remote (triangle) biopsies and no clear clusters of patients. **d:** Gene Ontology analysis demonstrating no significant differential expression of oxidative phosphorylation genes in ventricular cardiomyocytes between segments with or without inducible ischaemia. ADP = adenosine diphosphate; ATP = adenosine triphosphate; CABG = coronary artery bypass grafting; CAD = coronary artery disease; CMR = cardiovascular magnetic resonance; LV = left ventricular; vCM = ventricular cardiomyocyte.

This study integrated phenotyping by CMR with LV tissue analysis to assess for persistent metabolomic and transcriptomic myocardial adaptations in patients with stable CAD and preoperative evidence of inducible ischemia. The central findings are as follows. (1) LV myocardium from patients with chronic CAD is energetically deficient as compared to non-failing human donor myocardium, despite increased expression of biological processes associated with OXPHOS. (2) There is no substantial regional difference in the energetic, metabolic or transcriptomic profile of myocardium from regions with or without inducible ischemia from the same LV. (3) Viable LV myocardium from patients with CAD and LV systolic impairment is energetically impaired with a different metabolic landscape (including increased levels of succinate) compared to myocardium from patients with CAD and preserved LVEF (Extended Data Fig. 1).

### Altered bioenergetic profile between stable CAD and donor myocardium

CAD is a leading global cause of morbidity and mortality in adults^10–12^. Although primarily a vascular disease, the downstream impact of CAD on ventricular structure and function can precipitate heart failure^13^. Despite advances in therapy, there is a need to improve outcomes in this large patient population, with growing consensus that future treatments need to target myocardial dysfunction directly^14^.

Analysis of the HEP data showed an increase in myocardial ADP, despite preservation of ATP, in patients with chronic CAD as compared to donor hearts leading to a lower ATP:ADP ratio, suggesting impaired energy availability. Accumulation of ADP is a marker of tissue ischemia^15^, driven by reduced ATP production and increased consumption, leading to disruption of the ATP:ADP ratio. Second, snRNA-seq highlighted multiple biological processes associated with OXPHOS that were upregulated in the myocardium of patients with CAD compared to heart donors, including upregulation of several genes encoding ETC complexes in multiple cell types. Integrating these results with the HEP data suggests that, in the presence of severe epicardial CAD, myocardial mitochondria fail to meet the bioenergetic needs of the cell and, thus, upregulate OXPHOS gene expression, a compensatory response aiming to increase energetic availability. However, as the key driver of pathophysiology is repetitive episodes of myocardial hypoxia, this increase in mitochondrial capacity does not translate to an improved ATP:ADP ratio, potentially because of the lack of oxygen and disrupted substrate utilization. Additionally, reduced expression of *SLC2A4*, the gene encoding the GLUT4 transporter, may signify a transition toward a fetal metabolic program, a finding previously reported in studies characterizing LV tissue from patients with heart failure^16,17^.

Finally, there was increased expression of the gene encoding MnSOD across multiple cell types in patients with CAD compared to controls. MnSOD is an important endogenous antioxidant, and the increased *SOD2* expression may be in response to augmented levels of mitochondrial reactive oxygen species (ROS). This may represent a potential therapeutic target in stable CAD, notably for mitochondria-targeted antioxidants.

### No large regional myocardial bioenergetic differences in stable CAD

On assessment of the regional bioenergetic profile of the patients with CAD, there was no significant difference in the myocardial ATP content or ATP:ADP ratio between regions with or without inducible ischemia, despite differences in MPR during preoperative adenosine stress CMR. These results align with animal models^18,19^ and prior human studies^20,21^, suggesting a lack of regional difference in myocardial energy availability in stable CAD. Furthermore, our broad metabolic profiling did not identify regional alterations in the bioenergetic landscape of patients with evidence of localized inducible ischemia. Notably, there was no substantial regional difference in the content of metabolites that accumulate in acute ischemia (for example, succinate, lactate and hypoxanthine)^2^. This can be explained as, although regions subtended by epicardial CAD are susceptible to intermittent episodes of altered perfusion during vasodilator stress, the metabolite imbalances seen in severe hypoperfusion are absent at rest. Additionally, in acute ischemia (that is, a different phenotype to the patients in this study), there is increased reliance on anaerobic glycolysis to generate ATP and maintain the ATP:ADP ratio. Conversely, we demonstrate that this branch of glucose metabolism is similar in segments with and without inducible ischemia, with no increase in glycolytic intermediates. Finally, the snRNA-seq data provided additional evidence that metabolism is similar between LV regions with and without inducible ischemia, including expression of genes encoding the ETC complexes, glucose metabolism (including GLUT1 and GLUT4 transporters) and bioenergetic regulation (including AMP-activated protein kinase and PGC-1α).

Integrating these findings with the control data suggests that, for patients with stable CAD, energetic impairment of the myocardium is a global, rather than a regional, phenomenon. These results provide further support for exploring therapies that target myocardial bioenergetic alterations in patients with CAD. Such therapies might complement coronary revascularization, as animal models show persistent abnormalities in myocardial mitochondrial function after restoration of epicardial blood flow^22,23^.

### Energetic impairment is greater in patients with impaired LV function

There is a paucity of research on myocardial metabolomic abnormalities in humans with CAD and mildly to moderately reduced LV contractile function. Stratifying the patient cohort by LV dysfunction, we demonstrated that patients with LV systolic impairment had a significant reduction in global ATP:ADP ratio as compared to patients with preserved LVEF. These results are consistent with previous studies showing decreased adenosine nucleotide concentrations in models of reduced LV function^5,6^ and support the paradigm that the failing heart is depleted of energy^24^. Additionally, metabolomic analysis revealed increased myocardial content of several metabolites when LV function was impaired. One metabolite, taurine, is implicated in the regulation of calcium homeostasis and reduction of oxidative stress^25^. Accumulation of myocardial taurine has been previously reported in preclinical models of ischemic LV dysfunction, potentially linked to reduced beta-alanine levels^26^. Future studies assessing the role of elevated taurine in models of ischemic heart failure models should be considered, notably to assess whether taurine acts as a cytoprotective agent through its action as an osmolyte.

We also found an increase in myocardial succinate in patients with LV dysfunction. Succinate accumulation is known to be a marker of acute ischemia, driven either by the reverse action of complex II in the ETC or its inhibition by a reduced CoQ pool in conjunction with glutaminolysis^2^. The accumulated succinate is rapidly oxidized on reperfusion, causing reverse electron transport at complex I and generation of pathological ROS^2^. Data on the role of succinate in the context of chronic ischemic heart disease and LV dysfunction are more limited. In animal models of heart failure secondary to transverse aortic constriction and myocardial infarction, myocardial succinate levels are increased compared to sham-operated mice or mice with compensated LV hypertrophy^27,28^. Our results align to these findings, suggesting elevated myocardial succinate levels in patients with CAD with reduced LVEF, potentially secondary to a degree of global tissue ischemia in the context of progressive LV modeling and increasing myocardial energy demands. Future studies assessing the role of myocardial succinate as a therapeutic target in patients with LV dysfunction should be explored.

Finally, our data also demonstrated decreased myocardial content of several acylcarnitines species in patients with reduced LVEF as compared to patients with preserved LV systolic function. This aligns with data in non-ischemic dilated cardiomyopathy (DCM), with a recent study suggesting low myocardial acylcarnitine and fatty acid levels in patients with advanced heart failure compared to control hearts, a finding potentially secondary to a defect of fatty acid import in DCM^29^.

### Limitations

First, we acknowledge that the CABG samples were exposed to a degree of ex vivo ischemia and degradation of metabolites, including HEPs, cannot be excluded. However, all samples were clamp-frozen in liquid nitrogen in cardiac theaters, within 60 s of being removed from the beating heart. Second, we acknowledge the limitations of using control donor myocardium in view of the inevitable ischemia that occurs after brainstem or circulatory death, tissue acquisition and sample processing. Notably, 10 of 11 (91%) of the control LV tissues were acquired from donation after brainstem death (DBD), with all biopsies in the metabolomic analyses occurring on the normoxic beating heart. Additionally, there are increasing data highlighting the stability of the transcriptome after short periods of cold ischemia^30^. The authors also recognize the lack of external control data for the LC–MS results. The paired biopsy technique, however, permits important analysis in human studies, controlling for the innate heterogeneity of patients with chronic CAD (for example, comorbidities and drug therapy). Fourth, despite meticulous attempts to ensure correct co-localization of the CMR data with the proposed biopsy site, the exact region of biopsy could not be definitively aligned with the CMR images. Fifth, the authors acknowledge that evidence of inducible myocardial ischemia on preoperative stress CMR does not equate to tissue hypoxia during episodes of hypoperfusion or confirm that patients experience repetitive episodes of transient myocardial ischemia; adenosine perfusion CMR was used as a surrogate marker of these scenarios. The utility of blood oxygen level-dependent (BOLD) CMR in future studies would be of value. Additionally, larger studies permitting robust integration of the metabolomic and transcriptomic data (notably in the reduced LVEF cohort) should be considered. Finally, not all experiments were performed on each patient, with relatively few myocardial samples undergoing snRNA-seq. The amount of tissue available was primarily driven by safety concerns, and, therefore, it was not possible to run all assays in each patient. This reflects the challenges of undertaking a beating-heart myocardial biopsy study in humans.

## Conclusions

Our study highlights the feasibility and utility of integrating advanced phenotyping by CMR with in-depth LV tissue analysis in patients with stable CAD. The results suggest that the myocardium of patients with CAD is energetically disrupted despite upregulation of OXPHOS pathways. Additionally, we demonstrate a lack of compelling regional difference in the LV metabolic and transcriptomic landscape of stable CAD, data that suggest global myocardial dysregulation of mitochondrial bioenergetics. Proof-of-mechanism trials of interventions that might improve myocardial energetics for patients with CAD (for example, mitochondria-targeted antioxidants), with or without concomitant coronary revascularization, should be considered.

## Methods

### Study overview

AMBITION, a single-center, observational cohort study, was approved by the National Research Ethics Service (NRES) Committee (East of England–Cambridgeshire and Hertfordshire; Regional Ethics Committee (REC) reference 19/EE/0166) and complied with the Declaration of Helsinki. Consecutive patients with CAD awaiting clinically indicated CABG were invited to participate and provide written informed consent between 2019 and 2021. Exclusion criteria for the study were: (1) valve disease requiring intervention during surgery; (2) evidence of concomitant non-ischemic cardiomyopathy; (3) liver failure (for example, international normalized ratio (INR) > 2); (4) contraindication to CMR; and (5) inability to give consent. Patients underwent preoperative CMR with quantitative stress perfusion and late gadolinium enhancement imaging. During CABG, transmural LV biopsies were acquired. Previously published data from donor LV tissue were used in the external control analysis^3,8^. The study workflow is summarized in Fig. 1a. A degree of selection bias cannot be excluded as suitability for myocardial biopsy was considered during the recruitment process.

### CMR protocol

Patients underwent CMR on a 1.5-T Siemens Aera scanner. Localizers were followed by balanced steady-state free precession (bSSFP) cine long-axis imaging. Intravenous adenosine was subsequently infused for 3–5 min at a rate of 140–210 µg kg^−1^ min^−1^ in a stepwise protocol, ensuring a symptomatic and hemodynamic response. Short-axis slices at the basal, mid-ventricular and apical levels were acquired during the first pass of a 0.5 mmol kg^−1^ intravenous (i.v.) gadobutrol bolus (Bayer). Automated perfusion maps were produced using inline software contained within the Gadgetron online reconstruction framework together with myocardial blood volume maps as described previously^31^. A top-up of 0.5 mmol kg^−1^ of i.v. gadobutrol was then administered, followed by bSSFP short-axis cine imaging. After 10 min, late gadolinium enhancement (LGE) images were acquired, followed by rest perfusion imaging^32^.

### CMR analysis

CMR analysis was performed after each scan to identify areas of inducible myocardial ischemia and provide volumetric and viability evaluation before surgery. The final CMR analysis was undertaken on CVI42 (Circle Cardiovascular Imaging) by an independent level-3 accredited operator blinded to the biopsy data. Biventricular volumetric analysis was performed, followed by calculation of ischemic myocardial burden and quality control of the automated quantitative perfusion results. A hypocontractile viable myocardial segment was defined as a hypokinetic or akinetic segment with ≤50% LGE transmurality. The extent of myocardial infarction was assessed by LGE quantification using the full width at half maximum technique^33^.

### CABG tissue collection

LV tissue from patients undergoing CABG was sampled from two or more pre-determined areas (as guided by CMR and coronary angiography data) on the beating heart during surgery: (1) a region of viable myocardium with inducible ischemia (‘ischemic’ biopsy); and (2) a region of remote myocardium with normal contractility, no qualitative evidence of inducible hypoperfusion on perfusion imaging and without infarct pattern LGE (‘remote’ biopsy). Each biopsy location was determined from analysis of the CMR and coronary angiograpy images, with co-localization performed in theater with the consultant cardiac surgeon. The a priori plan was to acquire paired biopsies, permitting HEP quantification and LC–MS for each individual patient. Surgical discretion, however, resulted in some patients not undergoing paired tissue collection. Conversely, where deemed safe, extra tissue was acquired for snRNA-seq. The CABG biopsies were performed on the beating heart (before aortic cross-clamp, cardioplegia and hypothermia in the cases using cardiopulmonary bypass) with a Tru-Cut needle or scalpel by the consultant cardiac surgeon. Most operations were performed off-pump without cardiopulmonary bypass. Samples were immediately clamp-frozen in theater using a Wollenberg clamp (manufactured by Josh Firman, LMB Workshop), which had been pre-cooled in liquid nitrogen until tissue acquisition. The clamps were then reopened, and the tissue rapidly transferred into Eppendorf tubes (pre-cooled in dry ice) before storage at −80 °C awaiting further analysis.

### Donor tissue acquisition

For the ATP and ADP analysis, LV biopsies were acquired from normoxic, beating human hearts at the time of DBD from donors who were considered unsuitable for cardiac transplantation^7^. There was no documented history of CAD in the donors, and all individuals died from intracranial hemorrhage. Informed consent was provided by a family member. This study was conducted under REC reference 15/EE/0152 (NRES Committee East of England–Cambridge South). The LV tissue for the snRNAseq data was acquired from DBD (*n* = 6) or donation after circulatory death (DCD) (*n* = 1) from individuals with an unremarkable cardiovascular history^8^. Tissue sample HCA_D11 was processed at the Wellcome Sanger Institute under REC reference 15/EE/0152 (NRES Committee East of England–Cambridge South). Tissue samples HCA_H2–H7 were processed at Harvard Medical School under Human Research Ethics Board approval Pro00011739 (University of Alberta). Informed consent was obtained from the donor families.

### Statistical analysis

Baseline characteristics for the patients with CAD are summarized as frequency (%) for categorical variables and mean (s.d., σ) or median (IQR) for continuous variables. The omic results are presented as mean (s.d., σ) or median (IQR) as appropriate. Arbitrary levels for the LC–MS metabolites are used to aid description and visualization.

The primary and secondary outcome measures included univariate and multivariable analyses to assess for a significant difference in the LV multiomic profile of (1) patients with CAD versus donor controls; (2) ischemic versus remote myocardium from within the same heart; and (3) patients with CAD with and without reduced LVEF. Further exploratory analysis was conducted assessing the metabolic profile of (1) patients with CAD with three or more LV hypocontractile viable segments compared to patients with fewer than three hypocontractile viable regions and (2) patients with CAD with a global MPR > 1.5 compared to patients with a global MPR ≤ 1.5. A full description of the statistical analyses is detailed in the tissue analysis sections below. Specifically, univariate analysis was performed using Welch’s *t*-test or paired *t*-test for normally distributed data and the Wilcoxon–Mann–Whitney *U*-test or Wilcoxon signed-rank test for non-normally distributed data. When comparing against donor control myocardium, the mean values from the ischemic and remote myocardium were used. The univariate HEP and metabolomic results are plotted without adjustment for multiple testing, aligning with prior metabolomic studies harnessing human LV tissue^4^. All other *P* values were adjusted for multiple testing using the Benjamini–Hochberg method. Analysis was performed in R (version 4.1) and Scanpy Jupyter Lab^34^ based on Python. Owing to the novelty of this multiomic human LV biopsy study, there were no prior utilizable data to a guide a power calculation. No formal sample size was, thus, calculated. As a steer toward what would represent a minimum number of samples to assess regional LV metabolic differences within patients, 30 patients provided 80% power when the probability that the ATP:ADP ratio is higher in the remote segment (compared to the ischemic segment) is at least 25% with a two-sided type I error rate of 0.05.

### Tissue analysis for HEPs

ATP and ADP concentrations were determined using a luciferase-based assay^35^. The protocol used in this experiment was previously published by our group, and segments of the following text have been recycled to ensure clarity^7^. Frozen LV samples (~1–5 mg) were homogenized in ice-cold perchloric acid extractant (3% v/v HClO_4_, 2 mM Na_2_EDTA, 0.5% Triton X-100). The supernatant was diluted to a concentration of 1 mg of frozen tissue per milliliter. Samples and ATP and ADP standards were pH neutralized using a potassium hydroxide solution (2 M KOH, 2 mM Na_2_EDTA, 50 mM MOPS), vortexed until the formation of a white precipitate (KClO_4_) and then subsequently centrifuged (17,000*g* for 1 min at 4 °C). For ADP measurements, 250 µl of neutralized sample supernatant was mixed with 250 µl of ATP sulfurylase assay buffer (20 mM Na_2_MoO_4_, 5 mM GMP, 0.2 U ATP sulfurylase (New England Biolabs)) and Tris-HCl buffer (100 mM Tris-HCl, 10 mM MgCl_2_ (pH 8.0)), with subsequent incubation for 30 min at 30 °C with shaking (500 r.p.m.), and finally heated at 100 °C for 5 min and then cooled on ice. Standards (100 µl), samples for ATP measurement (100 µl) or samples for ADP measurement (200 µl) (in duplicate) were added to 400 µl of Tris-acetate (TA) buffer (100 mM Tris, 2 mM Na_2_EDTA, 50 mM MgCl_2_, pH 7.75 with glacial acetic acid) in luminometer tubes. Then, 10 µl of pyruvate kinase solution (100 mM PEP and 6 U of pyruvate kinase suspension (Sigma-Aldrich, P1506)) was added to one set of samples for ADP measurement and incubated for 30 min at 25 °C in the dark to convert ADP to ATP. The additional duplicate tube (without addition of pyruvate kinase solution) served as an ADP ‘blank’ value. Next, all samples were then assayed for ATP content in a Berthold AutoLumat Plus luminometer LB953 by addition of 100 µl of Luciferase/Luciferin Solution (7.5 mM DTT, 0.4 mg ml^–1^ BSA, 1.92 µg of luciferase per milliliter (Sigma-Aldrich, L9506), 120 µM d-luciferin (Sigma-Aldrich, L9504), made in TA buffer (25% v/v glycerol)), delivered via auto injection and protected from light. Bioluminescence of the ATP-dependent luciferase activity was determined for 45 s after injection, and the results were quantified against standard curves.

The HEP data solely underwent univariate analysis (without adjustment for multiple testing). First, we assessed the HEP content of LV myocardium from patients with CAD compared to donor controls. In this experiment, the mean HEP values from the remote and ischemic samples in the patients with CAD were used. Analysis was performed using Welch’s *t*-test for normally distributed data and the Wilcoxon–Mann–Whitney *U*-test for non-normally distributed data. Second, we performed paired analysis of the HEP content in ischemic versus remote myocardium from within the same LV. Analysis was performed using the paired *t*-test for normally distributed data and the Wilcoxon signed-rank test for non-normally distributed data. Third, we assessed the LV HEP levels of patients with CAD with impaired LVEF compared to the patients with CAD with preserved LVEF. In this experiment, ischemic and remote biopsies were individually included as biological replicates. All analysis was conducted in the R environment (including the rstatix package), generating plots using ggplot2 (ref. ^36^) and ggsignif.

### Tissue extraction and metabolite analysis by LC–MS

The protocol used in this experiment was previously published by our group, and sections of the following text have been recycled to ensure clarity^37^. Frozen LV tissue samples (~1–5 mg) were weighed into Precellys tubes (Stretton Scientific), and an exact volume of extraction solution (50% methanol, 30% acetonitrile and 20% water) was added to obtain 40 mg of specimen per milliliter of extraction solution, permitting comparisons between experimental conditions for the same metabolite. The samples were subsequently lysed after the addition of three ceramic beads using a Precellys 24 tissue homogenizer (Bertin, 5,500 r.p.m for 15 s × 2) and finally centrifuged (16,162*g* for 10 min at 4 °C). The supernatant was transferred into glass vials (MicroSolv Technology) and stored at −80 °C until LC–MS analysis.

HILIC chromatographic separation of metabolites was achieved using a Millipore SeQuant ZIC-pHILIC analytical column (5 µm, 2.1 × 150 mm) equipped with a 2.1 × 20-mm guard column (both 5-mm particle size) with a binary solvent system. Solvent A was 20 mM ammonium carbonate and 0.05% ammonium hydroxide; Solvent B was acetonitrile. The column oven and autosampler tray were kept at 40 °C and 4 °C, respectively. The chromatographic gradient was run at a flow rate of 0.200 ml min^−1^ as follows: 0–2 min: 80% B; 2–17 min: linear gradient from 80% B to 20% B; 17–17.1 min: linear gradient from 20% B to 80% B; 17.1–22.5 min: hold at 80% B. Samples were randomized and analyzed with LC–MS in a blinded manner with an injection volume of 5 µl. Pooled samples were generated from an equal mixture of all individual samples and analyzed interspersed, at regular intervals, within the sample sequence as a quality control. Each sample was analyzed with three analytical replicates.

Metabolites were measured using a Thermo Fisher Scientific Q Exactive Hybrid Quadrupole-Orbitrap Mass Spectrometer (HRMS) coupled to a Dionex UltiMate 3000 UHPLC. The mass spectrometer was operated in full-scan, polarity-switching mode, with the spray voltage set to +4.5 kV/−3.5 kV, the heated capillary held at 320 °C and the auxiliary gas heater kept at 280 °C. The sheath gas flow was programmed to 55 units; the auxiliary gas flow was programmed to 15 units; and the sweep gas flow was programmed to 0 units. HRMS data acquisition was performed in a range of *m/z* = 70–900, with the resolution set at 70,000, the AGC target at 1 × 10^6^ and the maximum injection time at 120 ms. Metabolite identities were confirmed using two parameters: (1) precursor ion *m*/*z* was matched within 5 p.p.m. of theoretical mass predicted by the chemical formula; and (2) the retention time of metabolites was within 5% of the retention time of a purified standard run with the same chromatographic method. Each sample underwent three analytical repeats with subsequent peak annotation, and chromatogram review and peak area integration were performed using Thermo Fisher Scientific TraceFinder 5.0 software. The peak area for each detected metabolite was subjected to the ‘Filtering 80% Rule’, half minimum missing value imputation, and normalized against the total ion count (TIC) to correct any variations introduced from sample handling through instrument analysis. Samples were excluded after performing testing for outliers based on geometric distances of each point in the PCA score analysis as part of the muma package (version 1.4)^38^.

The normalized LC–MS results were first explored by PCA to assess for clusters within the patients with CAD, with subsequent labeling of the ischemic and remote samples. PCA analysis was performed using the R base package stats (version 4.0.5) (https://www.r-project.org/) with the function prcomp and visualized using the autoplot function of ggplot2 (version 3.3.5)^36^ after loading the ggfortify package (version 0.4.14)^39^. Next, differential metabolite analysis was performed to investigate patterns of metabolite changes within patients (for example, between ischemic and remote samples) that were consistent across the cohort. Differential metabolomics analysis was performed using the R package gtools (version 3.9.2)^40^ to calculate the log_2_FC using the functions foldchange and foldchange2logratio (parameter base = 2). The corresponding *P* value was calculated using the R base package stats (version 4.1.3) with the functions wilcox.test or t.test and adjusted using the Benjamini–Hochberg method. Volcano plots were generated using the EnhancedVolcano package (version 1.12.0)^41^ and correlation plots using corrplot (version 0.92). Detailed code can be found at https://github.com/ChristinaSchmidt1/AMBITION_study. Univariate analysis of specific metabolites of interest was conducted, comparing ischemic and remote segments. In this experiment, statistical analysis was conducted on the mean values of the analytical repeats, and no adjustment for multiple testing was plotted. Analysis was performed using the paired *t*-test for normally distributed data and the Wilcoxon signed-rank test for non-normally distributed data. Finally, we performed exploratory analysis assessing the LV metabolic landscape of (1) patients with CAD with impaired LVEF compared to patients with CAD with preserved LVEF; (2) patients with CAD with three or more LV hypocontractile viable segments compared to patients with fewer than three hypocontractile viable regions; and (3) patients with CAD with a global MPR > 1.5 compared to patients with a global MPR ≤ 1.5. In these experiments, ischemic and remote biopsies were individually included as biological replicates. Analysis was performed using Welch’s *t*-test for normally distributed data and the Wilcoxon–Mann–Whitney *U*-test for non-normally distributed data.

### Single-nucleus preparation and RNA sequencing

Clamp-frozen myocardial samples (~3–5 mg) were processed for nuclei isolation and snRNA-seq as previously described^8,42^. In brief, samples were mechanically digested using dounce homogenization, and nuclei were sorted based on NucBlue (Thermo Fisher Scientific) positive staining using the FACSAria Fusion Cell Sorter (BD Biosciences). Sorted nuclei were visually inspected under microscope to assess integrity and manually counted using a hemocytometer. Nuclei were loaded onto the Chromium Controller (10x Genomics), and, in view of the limited mass of each biopsy, 500 nuclei were targeted per reaction. 3′ gene expression libraries were generated using Chromium Next GEM Single Cell v3.1 3′ GEX kits (10x Genomics) as per the manufacturer’s instructions. Quality control of cDNA and libraries was performed using Bioanalyzer High Sensitivity DNA Analysis (Agilent). Sequencing was performed using NovaSeq (Illumina) at a sequencing depth of 50,000 reads per nuclei. The raw sequence reads were mapped to the human reference genome (GRCh38 v2020-A) provided by 10x Genomics and using the CellRanger suite (version 5.0.1) with default parameters recommended for single-nucleus samples. Data for control samples were extracted from the healthy heart atlas study^8^, taking samples that came from the LV free wall and used V3 10x chemistry to match the region and chemistry used for the CABG samples. The snRNA-seq data were processed using the Scanpy toolkit (version 1.8.2)^12^, using the quality control thresholds and approach previously described^8^. Batch correction was performed using Harmony^43^ to correct for patient variation. Cell type annotation was performed by looking for enrichment of previously described marker genes in specific clusters^8^. Differentially expressed genes between (1) patients with CAD and controls and (2) ischemic and remote myocardial segments were compared by converting snRNA-seq counts to pseudobulk per sample and per cell type, followed by analysis using edgeR^44^. Enrichment of biological process Gene Ontology terms was performed using ShinyGO (version 0.75)^45^, with plots created in R using ggplot2. *P* values were adjusted for multiple testing using false discovery rate (FDR). Due to the limited number of samples, analysis assessing for differentially assessed genes between patients with CAD with and without reduced LVEF could not be performed.

### Reporting summary

Further information on research design is available in the Nature Portfolio Reporting Summary linked to this article.

### Supplementary information

**Extended Data Fig. 2:**
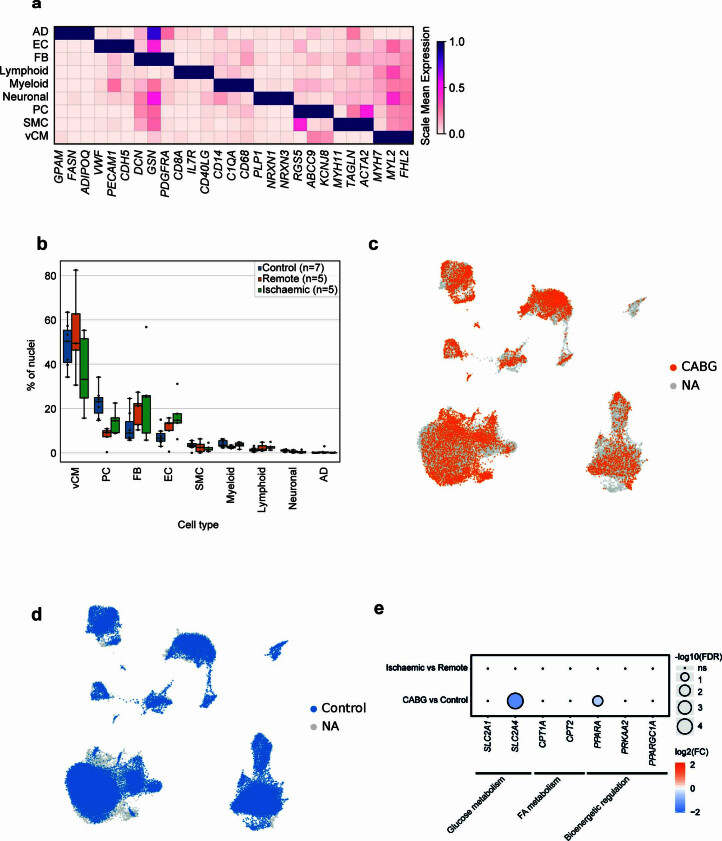
Overview of the snRNAseq data and additional analyses. **a**. Heatmap showing the specificity of marker genes used to annotate the cell type label for each cluster. **b**. Boxplot showing the abundance of each cell type per sample for each group, calculated from snRNAseq data. **c**. UMAP embedding of all nuclei highlighting those from CABG patients. **d**. UMAP embedding of all nuclei highlighting those from controls. **e**. Analysis of specific genes implicated in glucose transport, fatty acid metabolism and bioenergetic regulation between ischemic and remote samples (ventricular cardiomyocytes only). CABG versus control sample results included as a reference. The results demonstrate no significant differential expression in the selected genes between regions. AD = adipocytes; CABG = coronary artery bypass grafting; EC = endothelial cells; FB = fibroblasts; FA= fatty acid; FC = fold change; FDR = false discovery rate; NA = not applicable; ns = non-significant; SMC = smooth muscle cells; snRNAseq = single-nuclei ribonucleic acid sequencing; PC = pericytes; vCM = ventricular cardiomyocytes.

**Extended Data Fig. 3:**
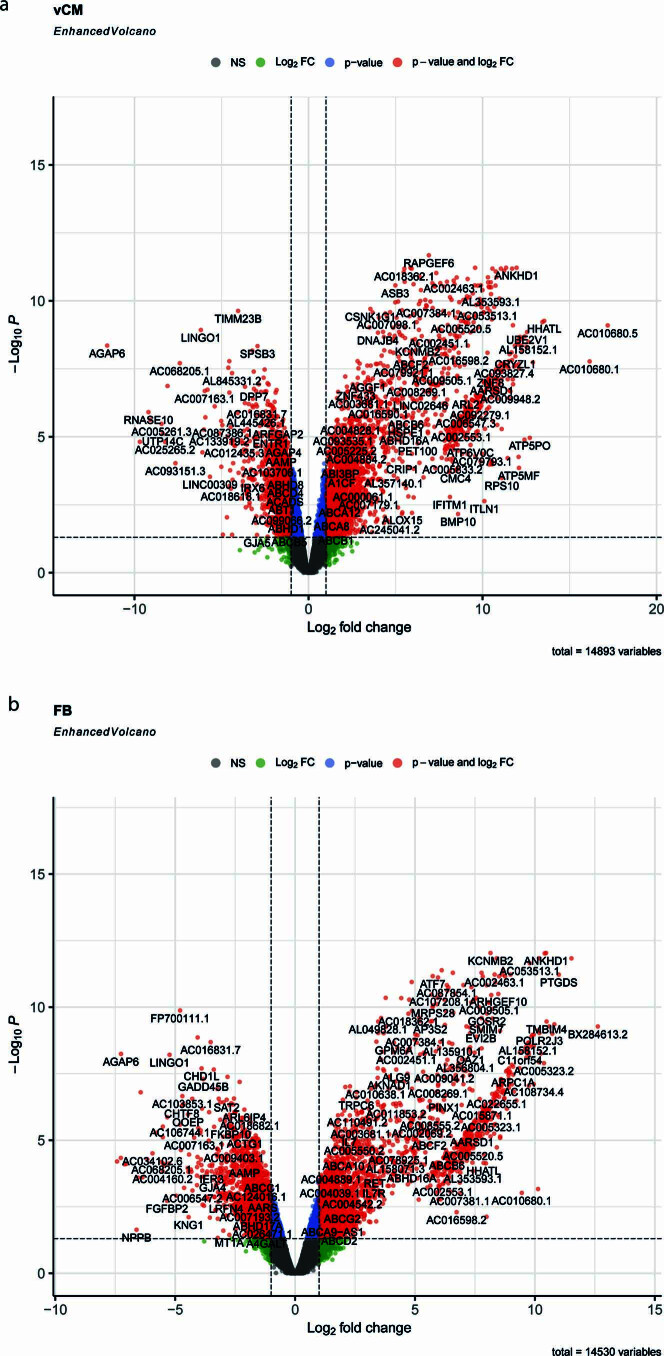
Volcano plot of snRNAseq data. Volcano plot detailing the differential gene expression within ventricular cardiomyocytes **(a)** and fibroblasts **(b)**, CABG versus controls. FB = fibroblasts; FC = fold change; NS = non-significant; vCM = ventricular cardiomyocyte.

**Extended Data Fig. 4:**
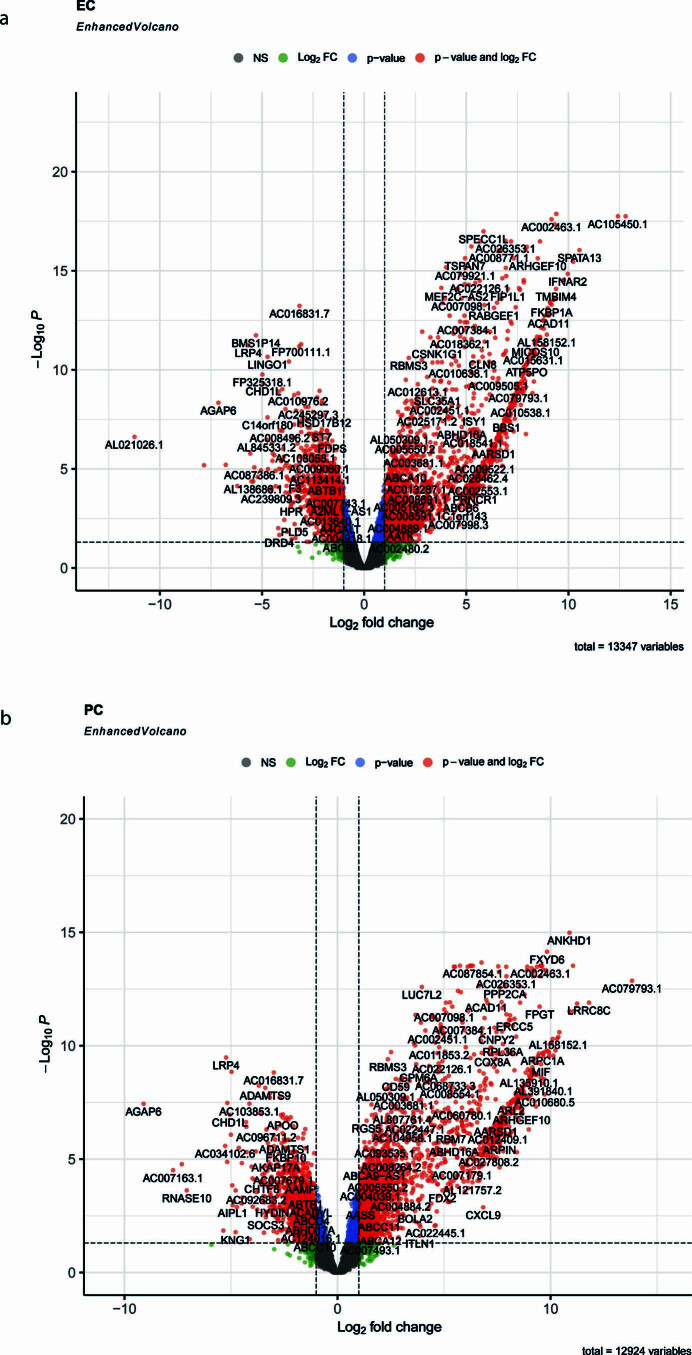
Volcano plot of snRNAseq data. Volcano plot detailing the differential gene expression within endothelial cells **(a)** and pericytes **(b)**, CABG versus controls. EC = endothelial cells; FC = fold change; NS = non-significant; PC = pericytes.

**Extended Data Fig. 5:**
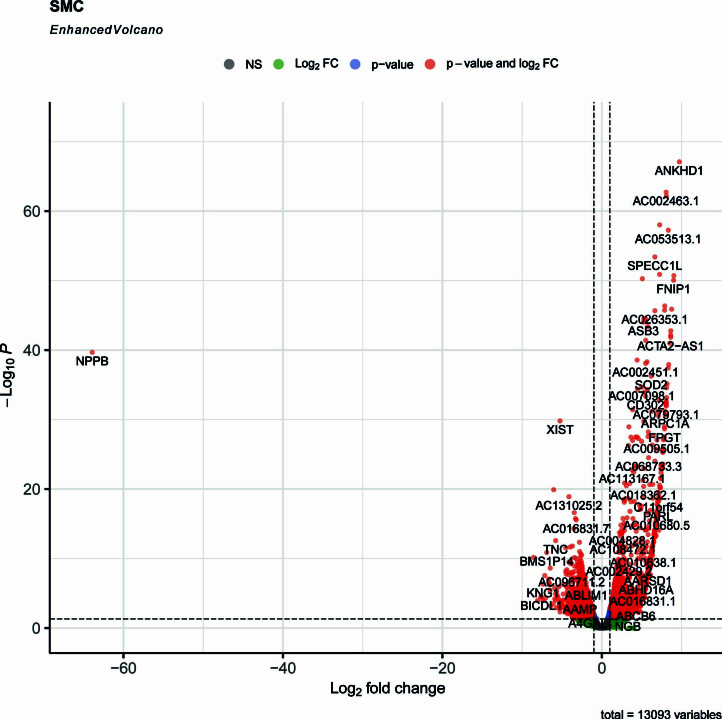
Volcano plot of snRNAseq data. Volcano plot detailing the differential gene expression within smooth muscle cells, CABG versus controls. FC = fold change; NS = non-significant; SMC = smooth muscle cells.

**Extended Data Fig. 6:**
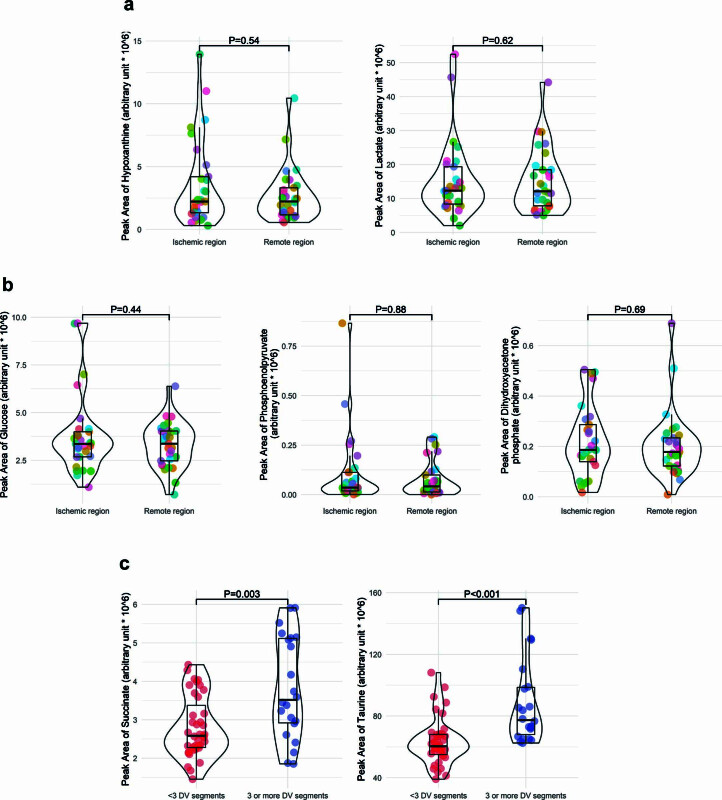
Supplementary univariate metabolomic results. **a**. Violin box plots detailing paired univariate analysis of myocardial hypoxanthine and lactate content (assessed by LC-MS) in chronically ischemic versus remote LV regions from the same heart. The results demonstrating no significant difference in the levels of these metabolites between regions**. b**. Violin box plots detailing paired univariate analysis of myocardial glucose content and glycolytic intermediate levels (assessed by LC-MS) in chronically ischemic and remote LV regions in the same heart. The results demonstrating no significant difference in the levels of these metabolites between regions. **c**. Exploratory univariate analysis of the LC-MS data in 29 patients, the results suggesting elevated succinate and taurine levels in CAD patients with ≥3 dysfunctional viable segments (that is, hypocontractile region with ≤50% transmurality of late gadolinium enhancement) compared to patients with <3 dysfunctional viable segments. The boxplots visualise the median value, in addition to hinges corresponding to the first and third quartiles, and whiskers corresponding to the largest/smallest value no further than 1.5*IQR from the hinge. Analysis was conducted using the Wilcoxon-signed-rank test. LC-MS = Liquid chromatography–*mass spectrometry;* LV = left ventricular.

**Extended Data Fig. 7:**
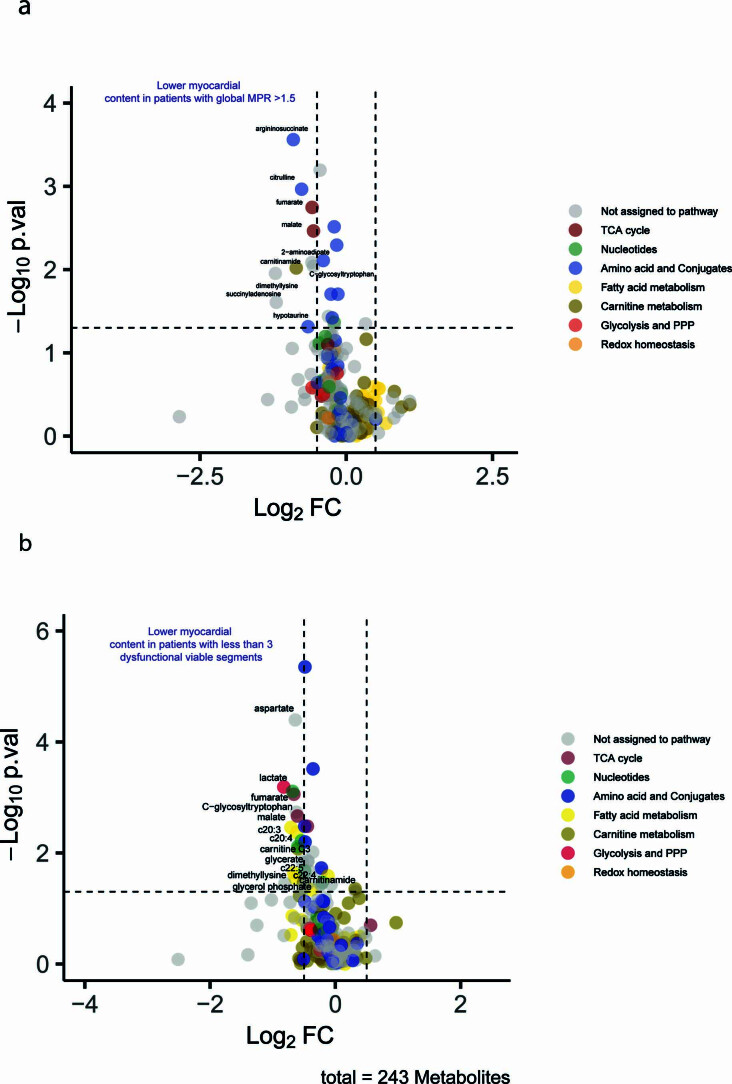
Supplementary volcano plots of the LC-MS data. **a**. Volcano plot of the LC-MS data highlighting metabolites that were significantly altered between patients with and without global MPR < 1.5; P-value of <0.05 with log2FC > 0.5 or log2FC < −0.5. **b**. Volcano plot of the LC-MS data highlighting metabolites that were significantly altered between patients with and without 3 or more dysfunctional viable LV segments (that is hypokinetic/akinetic segment with <50% LGE transmurality); P-value of <0.05 with log2FC > 0.5. FC = fold change; LC-MS = Liquid chromatography–*mass spectrometry; LGE = late gadolinium enhancement; LV = left ventricular; MPR = myocardial perfusion reserve*.

Supplementary Information Supplementary Materials, including Extended Data Tables 1–3 and Extended Data Figs. 1–7

Reporting Summary

Supplementary Table Additional metabolomic and snRNA-seq data

## Figures and Tables

**Extended Data Table 1:**
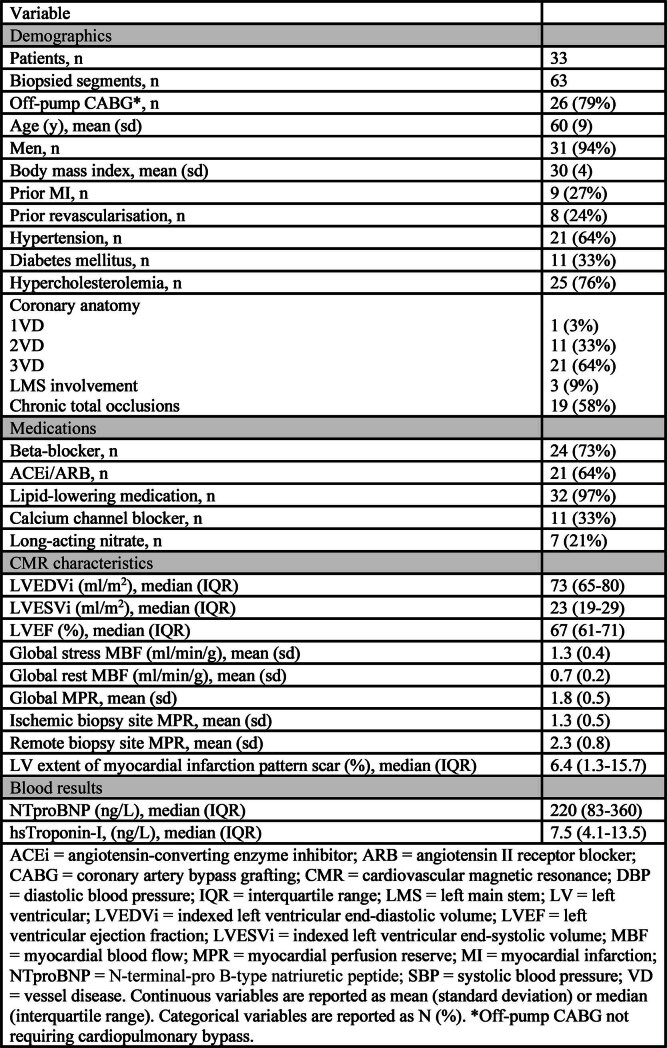
Baseline characteristics of the patients undergoing CABG

ACEi, angiotensin-converting enzyme inhibitor; ARB, angiotensin II receptor blocker; DBP, diastolic blood pressure; LMS, left main stem; LVEDVi, indexed left ventricular end-diastolic volume; LVESVi, indexed left ventricular end-systolic volume; MBF, myocardial blood flow; MI, myocardial infarction; NTproBNP, N-terminal-pro B-type natriuretic peptide; SBP, systolic blood pressure; VD, vessel disease. Continuous variables are reported as mean (s.d.) or median (IQR). Categorical variables are reported as *n* (%). * Off-pump CABG not requiring cardiopulmonary bypass.

**Extended Data Table 2:**
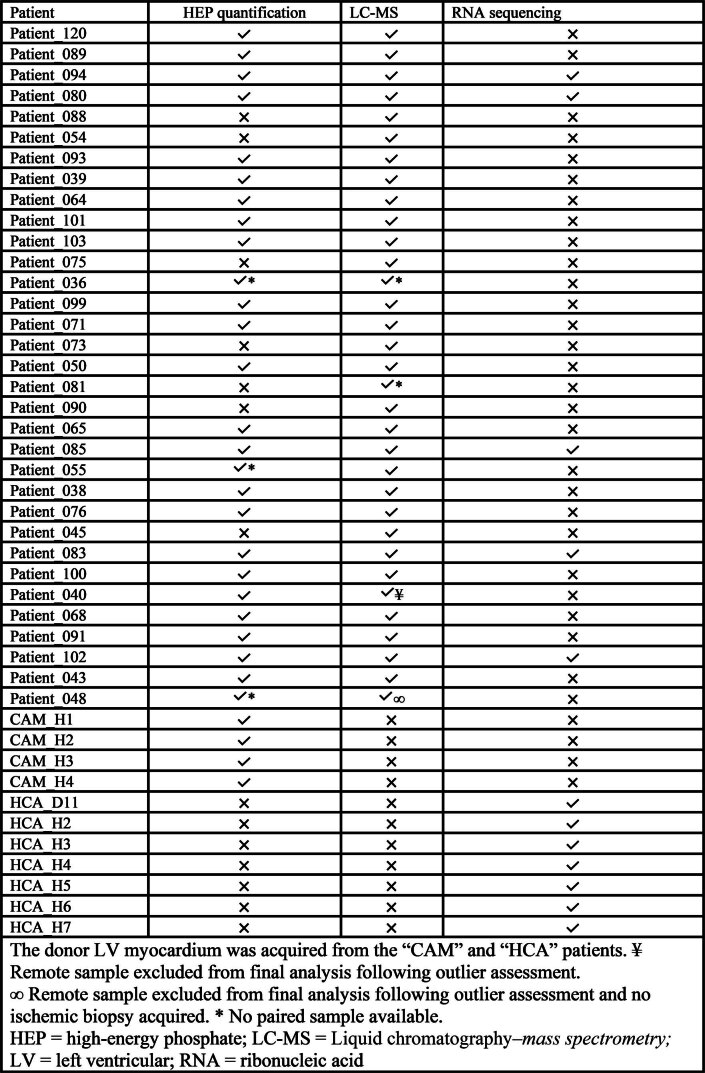
Breakdown of the experiments performed on each patient

The donor LV myocardium was acquired from the ‘CAM’ and ‘HCA’ patients. ¥ Remote sample excluded from final analysis after outlier assessment. ∞ Remote sample excluded from final analysis after outlier assessment and no ischemic biopsy acquired. * No paired sample available.

**Extended Data Table 3:**
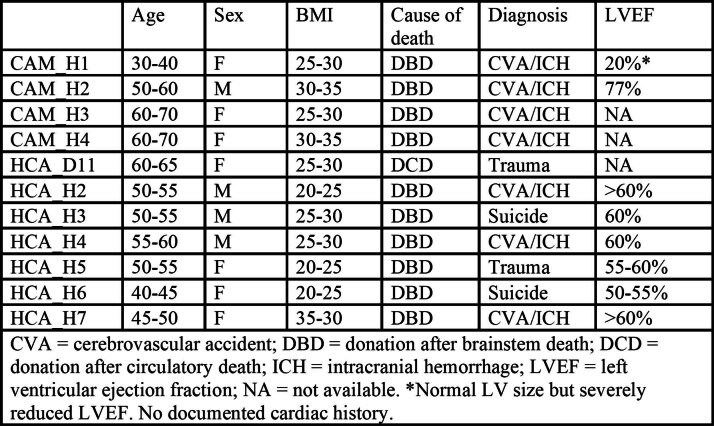
Metadata for the donor control myocardium

CVA, cerebrovascular accident; ICH, intracranial hemorrhage; NA, not available. * Normal LV size but severely reduced LVEF. No documented cardiac history.

## Data Availability

The metabolomic data are presented in the Supplementary Data Table and are deposited on Metabolomics Workbench^46^ under accession number ST002736. All sequencing data generated and analyzed here have been deposited at the European Genome-phenome Archive under accession number EGAS00001007351 and are available upon reasonable request.

## Code availability

The detailed code for the metabolomic analysis can be found at https://github.com/ChristinaSchmidt1/AMBITION_study. All code used to analyze snRNA-seq data can be found at https://github.com/NosedaLab/AMBITION_study.
