# Supplementary material for: Assessment of left ventricular tissue mitochondrial bioenergetics in patients with stable coronary artery disease
Source: Nat Cardiovasc Res. 2023 Aug 7;2(8):733–45. doi: 10.1038/s44161-023-00312-z (PMC11041759; doi:10.1038/s44161-023-00312-z)
Supplement: Supplementary file 1 — Supplementary Materials, including Extended Data Tables 1–3 and Extended Data Figs. 1–7 [file 44161_2023_312_MOESM1_ESM.pdf]

nature cardiovascular research

Letter

https://doi.org/10.1038/s44161-023-00312-z

# Assessment of left ventricular tissue mitochondrial bioenergetics in patients with stable coronary artery disease

In the format provided by the authors and unedited

# Supplementary materials

## Extended results

Overall, 32 (97%) patients underwent quantitative stress perfusion cardiovascular magnetic resonance (CMR); 30 patients had 3-slice quantitative stress perfusion during a research scan with 2 patients undergoing 6-slice quantitative perfusion during a clinical study. Additionally, 1 patient had failed injection of gadolinium contrast during the stress perfusion module.

A sensitivity analysis was conducted to check whether the CABG versus control HEP results were due to the inclusion of patients with reduced LV systolic function. In this analysis, only patients with normal LVEF according to the normal ranges adjusted for age, sex and body surface area were included (n=20).[1] In this analysis, there remained a significant difference in the LV ATP/ADP ratio between CAD patients and donors (median [IQR]: 2.6 [1.8-3.0] and 7.4 [6.8-8.6], respectively, P<0.001).

In the exploratory analysis, we additionally stratified patients by the i) presence or absence of 3 or more hypocontractile (but viable) LV segments and ii) global MPR on CMR. These results are detailed in Supplementary data table 1 and Extended Figures 6-7.

## Bibliography


1. Maceira, A. M., Prasad, S. K., Khan, M., & Pennell, D. J. (2006). Normalized left ventricular systolic and diastolic function by steady state free precession cardiovascular magnetic resonance. *Journal of Cardiovascular Magnetic Resonance : Official Journal of the Society for Cardiovascular Magnetic Resonance*, *8*(3), 417–426. https://doi.org/10.1080/10976640600572889
