# Supplementary material for: Assessment of left ventricular tissue mitochondrial bioenergetics in patients with stable coronary artery disease
Source: Nat Cardiovasc Res. 2023 Aug 7;2(8):733–45. doi: 10.1038/s44161-023-00312-z (PMC11041759; doi:10.1038/s44161-023-00312-z)
Supplement: Supplementary file 2 — Reporting Summary [file 44161_2023_312_MOESM2_ESM.pdf]

nature portfolio

Corresponding author(s): SK Prasad

Last updated by author(s): Jun 19, 2023

# Reporting Summary

Nature Portfolio wishes to improve the reproducibility of the work that we publish. This form provides structure for consistency and transparency in reporting. For further information on Nature Portfolio policies, see our Editorial Policies and the Editorial Policy Checklist.

## Statistics

For all statistical analyses, confirm that the following items are present in the figure legend, table legend, main text, or Methods section.

| n/a | Confirmed | |
|---|---|---|
| ☐ | ☒ | The exact sample size (*n*) for each experimental group/condition, given as a discrete number and unit of measurement |
| ☐ | ☒ | A statement on whether measurements were taken from distinct samples or whether the same sample was measured repeatedly |
| ☐ | ☒ | The statistical test(s) used AND whether they are one- or two-sided<br>*Only common tests should be described solely by name; describe more complex techniques in the Methods section.* |
| ☐ | ☒ | A description of all covariates tested |
| ☐ | ☒ | A description of any assumptions or corrections, such as tests of normality and adjustment for multiple comparisons |
| ☐ | ☒ | A full description of the statistical parameters including central tendency (e.g. means) or other basic estimates (e.g. regression coefficient) AND variation (e.g. standard deviation) or associated estimates of uncertainty (e.g. confidence intervals) |
| ☐ | ☒ | For null hypothesis testing, the test statistic (e.g. *F*, *t*, *r*) with confidence intervals, effect sizes, degrees of freedom and *P* value noted<br>*Give P values as exact values whenever suitable.* |
| ☒ | ☐ | For Bayesian analysis, information on the choice of priors and Markov chain Monte Carlo settings |
| ☒ | ☐ | For hierarchical and complex designs, identification of the appropriate level for tests and full reporting of outcomes |
| ☒ | ☐ | Estimates of effect sizes (e.g. Cohen's *d*, Pearson's *r*), indicating how they were calculated |

*Our web collection on statistics for biologists contains articles on many of the points above.*

## Software and code

Policy information about availability of computer code

| | |
|---|---|
| Data collection | Software used include: FACSAria Fusion Cell Sorter (BD Biosciences), Chromium Controller Firmware version 5.00-5.01 (10X Genomics), Thermo Fisher Tracefinder 5.0 |
| Data analysis | Software used include: R 4.1.2 and R 4.1.3, Python 3.9.7, 10X Genomics' CellRanger suite (v5.0.1), EdgeR (v. 3.32.1), harmonypy (v0.0.5), Scanpy toolkit (v1.8.2), ShinyGO (v0.75), ggplot2, EnhancedVolcano package (v. 1.12.0), gtools (v.3.9.2), ggfortify package (v.0.4.14), ggsignif, muma package (v.1.4), corrplot (v.0.92), Adobe Illustrator. The code of the metabolomics analysis has been deposited at https://github.com/ChristinaSchmidt1/AMBITION_study. |

For manuscripts utilizing custom algorithms or software that are central to the research but not yet described in published literature, software must be made available to editors and reviewers. We strongly encourage code deposition in a community repository (e.g. GitHub). See the Nature Portfolio guidelines for submitting code & software for further information.

## Data

Policy information about availability of data

All manuscripts must include a data availability statement. This statement should provide the following information, where applicable:

- Accession codes, unique identifiers, or web links for publicly available datasets
- A description of any restrictions on data availability
- For clinical datasets or third party data, please ensure that the statement adheres to our policy

There are no restrictions on data availability. The code of the metabolomics analysis can be found at https://github.com/ChristinaSchmidt1/AMBITION_study and the data has been deposited at Metabolomics Workbench under accession number ST002736. All sequencing data generated and analysed here has been deposited at the European Genome-Phenome Archive (EGA) under accession number EGAS00001007351 and is available upon reasonable request. All code used to analyse snRNAseq data can be found at https://github.com/NosedaLab/AMBITION_study.

## Human research participants

Policy information about studies involving human research participants and Sex and Gender in Research.

| | |
|---|---|
| Reporting on sex and gender | The biological sex for all participants is reported in the manuscript. |
| Population characteristics | All population characteristics are detailed in the manuscript and baseline demographic tables |
| Recruitment | Left ventricular biopsies were acquired on 33 prospectively recruited, consecutive, patients with stable coronary artery disease aged 47-77 undergoing coronary artery bypass grafting at a single UK Hospital Trust. Informed written consent was acquired on all patients. As safety was a key consideration, a degree of selection bias cannot be excluded as suitability for myocardial biopsy was considered during the recruitment process.<br><br>Control donor heart biopsies were acquired from deceased human DBD (n=10) and DCD (n=1) donors deemed unsuitable for cardiac transplantation. Informed consent for the use of the human tissue for this study was provided by the donors' families. |
| Ethics oversight | Ethical approval for the patients undergoing coronary artery bypass grafting was obtained from NRES Committee East of England - Cambridgeshire and Hertfordshire (REC Reference 19/EE/0166). Informed consent was acquired from the patients.<br><br>Ethical approval for the control donor tissue was obtained from NRES Committee East of England – Cambridge South (REC Reference 15/EE/0152) and Human Research Ethics Board approval Pro00011739 (University of Alberta, Edmonton, Canada). Informed consent from donor families. |

Note that full information on the approval of the study protocol must also be provided in the manuscript.

# Field-specific reporting

Please select the one below that is the best fit for your research. If you are not sure, read the appropriate sections before making your selection.

☒ Life sciences ☐ Behavioural & social sciences ☐ Ecological, evolutionary & environmental sciences

For a reference copy of the document with all sections, see nature.com/documents/nr-reporting-summary-flat.pdf

# Life sciences study design

All studies must disclose on these points even when the disclosure is negative.

| | |
|---|---|
| Sample size | Due to the novelty of this multiomic human LV biopsy study there was no prior utilisable data to a guide a power calculation. No formal sample size was thus calculated. As a steer towards what would represent a minimum number of samples to assess regional LV metabolic differences within patients, 30 patients provided 80% power when the probability that the ATP/ADP ratio is higher in the remote segment (compared to the ischaemic segment) is at least 25% with a two-sided type I error rate of 0.05. |
| Data exclusions | In the LC-MS analyis, samples were excluded after performing testing for outliers based on geometric distances of each point in the PCA score analysis as part of the muma package (v.1.4). No data from the high energy phosphate analysis was excluded.<br><br>For the single nuclei RNA sequencing, sorted nuclei were visually inspected under microscope to assess integrity and manually counted using a haemocytometer. Nuclei were loaded onto the Chromium Controller (10x Genomics), and in view of the limited mass of each biopsy, 500 nuclei were targeted per reaction. |
| Replication | For the majority of patients, 2 samples from differing regions in the LV were acquired. Specifically for the LC-MS analysis, after extraction, each sample was measured 3 times (analytical replicates) with good success. Data from 5 CAD patient hearts and 7 control hearts was used in the single nuclei RNA sequencing analysis, with comparable results among all the hearts within each group. |

| | |
|---|---|
| Randomization | In the LC-MS experiment, all samples were run together in a randomised pattern. Otherwise, randomisation was not relevant for this study as the samples were analysed in consecutive order based on availability of the myocardial tissue |
| Blinding | All samples were linked-anomymised prior to analysis. |

# Reporting for specific materials, systems and methods

We require information from authors about some types of materials, experimental systems and methods used in many studies. Here, indicate whether each material, system or method listed is relevant to your study. If you are not sure if a list item applies to your research, read the appropriate section before selecting a response.

## Materials & experimental systems

| n/a | Involved in the study |
|---|---|
| ☒ | ☐ Antibodies |
| ☒ | ☐ Eukaryotic cell lines |
| ☒ | ☐ Palaeontology and archaeology |
| ☒ | ☐ Animals and other organisms |
| ☐ | ☒ Clinical data |
| ☒ | ☐ Dual use research of concern |

## Methods

| n/a | Involved in the study |
|---|---|
| ☒ | ☐ ChIP-seq |
| ☒ | ☐ Flow cytometry |
| ☒ | ☐ MRI-based neuroimaging |

# Clinical data

Policy information about clinical studies

All manuscripts should comply with the ICMJE guidelines for publication of clinical research and a completed CONSORT checklist must be included with all submissions.

| | |
|---|---|
| Clinical trial registration | AMBITION was an observational cohort study and does not meet the ICMJE criteria for a clinical trial. AMBITION was thus not registered on a clinical trial site |
| Study protocol | The full clinical study protocol can be requested from the corresponding author |
| Data collection | Patients undergoing coronary artery bypass grafting were recruited from a single UK Hospital Trust between September 2019-April 2021 |
| Outcomes | The multiomic profile of: i) left ventricular tissue from patients with stable coronary artery disease versus control donor left ventricular tissue ii) left ventricular tissue from regions with and without inducible ischemia in patients with stable coronary artery disease; iii) left ventricular tissue from stable coronary artery disease patients with and without left ventricular systolic impairment |
